# Gemcitabine cationic polymeric nanoparticles against ovarian cancer: formulation, characterization, and targeted drug delivery

**DOI:** 10.1080/10717544.2022.2058645

**Published:** 2022-04-01

**Authors:** Sankha Bhattacharya, Md Meraj Anjum, Krishna Kumar Patel

**Affiliations:** a Department of Pharmaceutics, School of Pharmacy & Technology Management, SVKM'S NMIMS Deemed-to-be University, Shirpur, Maharashtra, India; b Department of Pharmaceutical Engineering & Technology, Indian Institute of Technology (Banaras Hindu University), Varanasi, Uttar Pradesh, India

**Keywords:** Gemcitabine, polysarcosin, OVCAR-8, EGFR vIII, confocal microscopy

## Abstract

This study focused on gemcitabine (GTB) delivery of cationic polymeric nanoparticles to treat ovarian cancer in order to promote effective localized delivery and drug retention during biological discharge. To begin, four GTB-loaded polymer nanoparticles were prepared: chitosan nanoparticles (CS-NPs), polysarcosin nanoparticles (PSar-NPs), poly-l-lysine & polysarcosin nanoparticles (PLL-PSar-NPs), and chitosan & polysarcosin nanoparticles (CS-PSar-NPs). Based on preliminary particle size, zeta potential, encapsulation efficiency, DSC, surface morphology, release profiling, and cellular internalization studies using rhodamine 123 and Nile red fluorescent markers, it was hypothesized that CS-PSar-NPs could be the best cationic formulation with strong biocompatibility and anticancer activity against the OVCAR-8 ovarian cancer cell line. To improve effective targeting, cellular penetration, and *in vitro* cytotoxicity, epidermal growth factor receptor variation III (EGFRvIII) is attached over all four polymeric nanoparticles. Confocal imaging revealed that EGFRvIII-conjugated cationic GTB polymeric nanoparticles had a greater cellular uptake and double internalization capabilities than unconjugated nanoparticles, as well as time-dependent cell entrance. GTB and EGFRvIII-conjugated polymer nanoparticles would have a stronger potential to infiltrate ovarian cancer cells during the first hour of incubation. According to TEM and FTIR findings, EGFRvIII conjugation across the non-target CS-PSar-NP surface was successful, making CS-PSar-NPS-EGFRvIII more target-specific and thus a safer drug delivery candidate for ovarian cancer treatment.

**Highlights**
- GTB loaded non-target CS-PSar-NPs & active targeted CS-PSar-NPs-EGFRvII developed.
- SEM, AFM, DSC, particle size, zeta potential, internalization performed for CS-PSar-NPs.
- MTT & CLSM study confirmed CS-PSar-NPS-EGFRvII was binding specific to OVCAR-8 cells
- Fabrication of EGFRvII over nanoparticles confirmed by TEM.
- CS-PSar-NPS-EGFRvII safer candidate for ovarian cancer.

## 1. Introduction

Ovarian cancer originates in the female eggs and often goes undetected unless it proliferates in the stomach and pelvis (Pitre, 2010; Mendes et al., 2021). These types of malignancies among women are challenging to treat, and therefore they are very much incurable (Gatenby & Brown, 2020; Shen et al., 2020; Annaji et al., 2021). It is the second most common gynecological cancer in females, with a registered incidence of about 15 cases per 100,000 women in western countries(Joachim et al., 2020). Early menopause, heavy consumption of saturated fats and refined carbohydrates intensively increase the risk of ovarian cancer (Cazzaniga et al., 2021). However, vegetable consumptions reduce the risk of ovarian carcinoma. Family history and environmental factors are very critical to diagnose the origin of ovarian cancer (Fu et al., 2021). As per the International Federation of Obstetrics and Gynecology (FIGO), cytoreductive surgery does not remain that much productive, as chemotherapy was found more effective to exterminate malignant foci (Charkhchi et al., 2020; Shabir & Gill, 2020). However, persistent side effects of the chemotherapeutic drug due to non-selectivity and non-site-specific release create an obstacle (Yokoe et al., 2021). As per the Ovarian Cancer Research Alliance (OCRA), 10% recurrence (were, same cancer cells left behind after treatment to shows apparent) observed if cancer is diagnosed in stage 1. However, almost 70% of ovarian cancer patients will have a recurrence if diagnosed in stage 2. To dodge such challenges, immunotherapy or intervascular therapy would be a good option (Zhang et al., 2015). The most commonly used intravesical and chemotherapeutic agents for acute ovarian cancer are Bevacizumab, Gemcitabine (GTB) Hydrochloride, Niraparib Tosylate Monohydrate, Olaparib, Rucaparib Camsyla. To maintain higher drug concentration on tumor site, to operate the drug exposure time, and to minimize systematic cytotoxicity by reducing systematic availability, intravesical chemotherapy and immunotherapy can play a pivotal role in ovarian tumor therapy. However, due to sustainable ovulation, endometrial secretion, and menstrual cycle, intravesical drug delivery is quite challenging because of frequent secretion (Raval et al., 2021; Subhan & Torchilin, 2021b). This might cause the failure of intravesical drug delivery (Feng & Chau, 2018) with chemotherapeutic medicaments, and therefore, it could potentiate the recurrence rate of cancer in the ovary (Rostamizadeh & Torchilin, 2020). To evade such problems, bio-adhesive colloidal drug delivery could be the best alternative for intravesical chemotherapeutic agents (Paciotti et al., 2004; Liang et al., 2021). In recent times, as per Abhijit A. Date et al. (2020) research, using polyethylene glycol (PEG), Pluronic F127 (Poloxamer 407), PEG660-12-hydroxy stearate, intravesical docetaxel nanosuspension can be prepared for the treatment of non-muscle-invasive bladder cancer (Bannigan et al., 2021; Subhan & Torchilin, 2021a). In the same pattern as per Ting-Yu Chen et al (2020), for the intravesical chemotherapy delivery of GTB via viscous nanoemulsion, formulations were prepared using capryol 90 (propylene glycol monocaprylate), 1,5-pentanediol, methylcellulose, benzalkonium chloride, tween 80 (Chen et al., 2020). As per Brandhonneur et al. (2018), for the treatment of ovarian cancer, molybdenum cluster-loaded PLGA nanoparticles were prepared by considering different synthesized salt of molybdenum hexanuclear i.e. TMB, CMB & CMIF, and in the presence of 0.5% *w/v* aqueous polysorbate 20 solutions (Brandhonneur et al., 2018). The nanoparticles were prepared while maintaining 500 rpm magnetic stirring at room temperature. These designs would certainly improvise the efficacy of intravesical chemotherapy.

In this research work, GTB was considered as a model drug. GTB inhibits ribonucleotide reductase, DNA, and thymidylate synthesis, and therefore, cancerous cells cannot copy host DNA, and hance dies. As per Deep Pooja et al. (2015) research work, overtrain cancer can be cured by GTB-loaded cyclic RGDfK peptide-functionalized polymeric nanoparticles; where 0.25% *w/v* Pluronic F68 was considered as a surfactant, 2 mL of PLGA solution (5 mg/mL in ethyl acetate) was considered as a polymeric solution (Pooja et al., 2015).

In this research work, an attempt was made to prepare cationic controlled release polymeric nano formulations encapsulated with GTB for intravesical chemotherapeutic agents, which could have a profile of higher drug concentration at the tumor site and could avoid drug loss during ovarian discharge. For the proper understanding of nanoparticle's behaviors, four types of GTB-loaded polymeric nanoparticles were prepared i.e. chitosan nanoparticles (CS-NPs), polysarcosine nanoparticles (PSar-NPs), poly-l-lysine & polysarcosine-fabricated nanoparticles (PLL-PSar-NPs), and finally, chitosan & polysarcosine-fabricated nanoparticles (CS-PSar-NPs).

All four formulations possessed cationic charges with different biological and physicochemical properties. These formulations were subjected to measurement of particle size & zeta potential, scanning electron microscopy (SEM), atomic force microscope (AFM), DSC analysis, encapsulation efficiency, *in vitro* drug release studies from nanoparticles.

The PSar-NPs were demonstrated as a major carrier for bioadhesive intravesical chemotherapy drug delivery. The study was plan to prepare and develop GTB encapsulated cationic nanoparticles carriers, which could be effective against ovarian cancer. However, CS-PSar-NPs was considered as one of the best formulations due to their biocompatibility, higher drug encapsulation efficacy, superior *in vitro* drug release profile, higher *in vitro* anticancer activity, and higher internalization behavior.

The molecular nature of tumor pathology was frequently detected, with overexpressed vascular endothelial growth factor (VEGF) (Guo et al., 2021) and some receptors on specific tumor surfaces. As a result, therapeutic anti-VEGF molecules may be a possible focus for those molecules. Cancer molecular targeting is critical for lowering dosage levels and improving formulation therapeutic efficacy.

**Figure 1.**
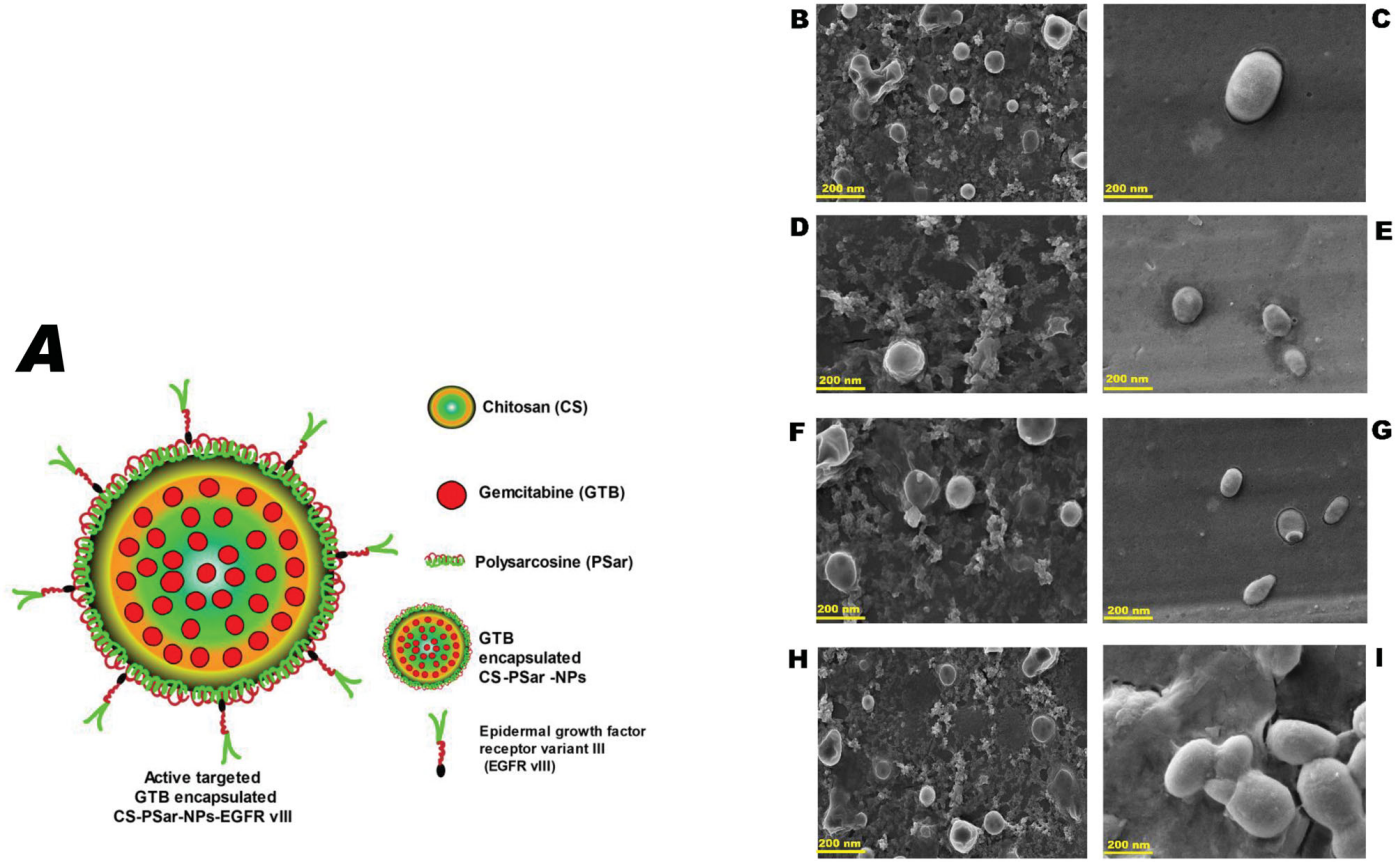
(A) Diagram presentation of chitosan (CS) core and polysarcosine (Psar) surface-coated epidermal growth factor receptor variant III (EGFR vIII) scaffold polymeric nanoparticles (CS-PSar-NPs-EGFRvIII) SEM photomicrographas of cationic nanoparticles prior to and following encapsulation, (B) blank CS nanoparticles, (C) gemcitabine-loaded CS-NPs, (D) blank PSar-NPs nanoparticles, (E) gemcitabine-loaded PSar-NPs, (F) blank PLL-PSar-NPs, (G) gemcitabine-loaded PLL-PSar-NPs, (H) Blank CS-PSar-NPs, and (I) gemcitabine-loaded CS-PSar-NPs.

Another objective of this research was also to focus on anchoring an anti-VEGF molecule, i.e. epidermal growth factor receptor variant III (EGFR vIII), on the surface of GTB encapsulated nanoparticles (CS-NPs, PSar-NPs, PLL-PSar-NPs, CS-PSar-NPs) to improve cellular uptake, active targeting efficiency, and *in vitro* cytotoxicity. In Figure 1(A), chitosan (CS) core and polysarcosine (Psar) surface-coated epidermal growth factor receptor variant III (EGFR vIII) scaffold polymeric nanoparticles (CS-PSar-NPs-EGFRvIII) were highlighted.

EGFRvIII-conjugated GTB nanoparticles (CS-NPs-EGFRvIII, PSar-NPs-EGFRvIII, PLL-PSar-NPs-EGFRvIII) and GTB-encapsulated nanoparticles all demonstrated *in vitro* cell viability in OVCAR-8 cells. Cellular uptake and specificity of EGFRvIII-conjugated nanoparticles were also tested using cell internalization, flow chamber assay for specific binding capacity in OVCAR-8 cells.

The fabrication of epidermal growth factor receptor variant III (EGFR vIII) on the surface of GTB-encapsulated polymeric nanoparticles, which improved cellular uptake in OVCAR-8 cells, was a novel aspect of this study. Most importantly, nanoparticles would retain in the ovary region due to their bio-adhesive-polymeric nature and survive ovulation, endometrial secretion, and hance such drug formulation would be the best candidate for intravesical drug delivery.

This research has also offered a foundation with technology that could be used to develop other therapeutic products. In the future, a xenograft mouse model will be developed for more research into ovarian cancer treatment.

## 2. Materials and methods

### 2.1. Materials

GTB was a gift sample from Neon Laboratories Ltd., Mumbai, India. EGFR Antibody (DH8.3)-EGFRvIII, Mutant (1 mg/ml) was a kind gift from Novus Biologicals, LLC (Toronto, Canada). Deacetylated chitosan (mol wt. 50,000–190,000 Da, with 75–85% deacetylation degree) (CS), polysarcosine (Psar), poly-l-lysine (PLL), rhodamine 123, Nile Red, triphenyl phosphine (TPP), polyvinyl alcohol (PVA) was purchased from Sigma Aldrich–Merck (Bengaluru, India). Pluronic^®^ F68, pH6.8 phosphate buffer (137 mM NaCl, 2.7 mM KCl, 10 mM Na_2_HPO_4_; 2 mM KH_2_PO_4_), MTT (3-(4,5-dimethylthiazol-2-yl)-2,5-diphenyltetrazolium bromide), fetal bovine serum (FBS) (endotoxin level: ≤5 EU/Ml; hemoglobin level: ≤15 mg/dL), sodium dodecyl sulfate (SDS) (>98% C12 alkyl sulfate with low levels of hexadecyl sulfate (C16)), Pierce™ dimethylsulfoxide (DMSO) (>99.5% pure), McCoy's 5 A (Modified) medium was purchased from ThermoFisher Scientific India Pvt Ltd, Navi Mumbai, India. The National Centre provided the human ovarian tumor cell line IGR-OV1, OVCAR-5, and OVCAR-8, Pune, India, for experimental uses; ELGA System (Woodridge, IL) was used to produce distilled water.

## 3. Preparation of cationic nanoparticles

### 3.1. Preparation of chitosan nanoparticles (CS-NPs)

To load GTB into bio-adhesive chitosan nanoparticles, the ionotropic gelation technique was implemented (Sacco et al., 2021). To obtain a blank polymeric nanoparticle, 0.7 mg/mL triphenylphosphine (TPP) solution has been added to 2.25 mg/mL of chitosan aqueous solution maintaining the temperature at 25 °C. The presence of TPP, which has a phosphorus center and is an active Lewis's base, could keep the reaction temperature stable. Nevertheless, TPP has sp^3^ hybridization at the phosphorous center; this molecule can able to donate another molecule, and hence, the coupling is believed to happen between the positive amino group of chitosan and negative TPP; which ultimately leads to prepare nanoparticles. To prepare GTB-loaded chitosan nanoparticles, GTB was dissolved into TPP solution, which was preloaded with 30% of chitosan (CS) solution. The protocol for preparing blank nanoparticles was subsequently followed. The prepared nanoparticles were washed three times at 15,000 rpm for 45 min with centrifugation. The nanoparticle pallets formed in the centrifuge tube bottom were further dispersed into distilled water and filtered with a membrane filter of 0.45 μm.

### 3.2. Preparation of polysarcosine nanoparticles (PSar-NPs)

The technique of nanoprecipitation was introduced to prepare polysarcosine nanoparticles. Where, with gentle heating, 15 mg of polysarcosine was dissolved into 10 mL of acetone. Under moderate magnetic stirring, the formulated polymeric solution was incorporated into 10 mL of ultrapure water comprising the required amount of GTB and Pluronic^®^ F68 (Costanzo et al., 2021). Further, the solution was sonicated for 15 min, and the organic solvent was evaporated under vacuum conditions at 37 °C to extract polymeric nanoparticles suspension of PSar-NPs.

### 3.3. Chitosan and polysarcosine fabricated nanoparticles (CS-PSar-NPs)

To prepare polysarcosine (PSar) nanoparticles, nanoprecipitation technique was implemented (Moreno-Vásquez et al., 2021). Where 15 mg of polysarcosine (Psar) was dissolved into 10 mL of acetone with gentle heating. The prepared polymeric solution was incorporated into 10 mL of ultrapure water containing Pluronic^®^ F68 under mild magnetic stirring. Under vacuum conditions, the organic solvent was evaporated to obtain uncoated polymeric nanoparticles. To produce chitosan and GTB-loaded polysarcosine (PSar) nanoparticles, chitosan (0.25% *w/v*) (CS), and GTB (15% of PSar weight) were dissolved in the aqueous phase. Further sonicated for 15 min and vacuum dried at 37 °C to obtain 8 mL drug-loaded chitosan and polysarcosine-fabricated nanoparticles (CS-PSar-NPs).

### 3.4. Poly-l-lysine and polysarcosine-fabricated nanoparticles (PLL-PSar-NPs)

The GTB-filled nanoparticles were first prepared with the nanoprecipitation technique to acquire PLL-PSar-NPs nanoparticles. GTB (15% polymer weight) was dissolved in an aqueous solution during the preparation of nanoparticles to achieve medication encapsulation in nanoparticles. GTB-loaded nanoparticles were then incubated for 45 min in a water solution of poly-l-lysine (PLL) (0.1% *v/v)* and centrifuged at 15,000 rpm to acquire coated nanoparticles after discarding the free-drug and free PLL containing supernatant. PLL-PSar-NPs nanoparticles in the precipitate were then further diluted into ultrapure water to obtain the final nanoparticles dispersion.

## 4. Characterization of cationic nanoparticles

### 4.1. Particle size distribution

Using Delsa Nano C instrument (Beckman Coulter, Carlsbad, CA, USA) (Pithanthanakul et al., 2021), the mean diameter (nm ± SD) and polydispersity index value of CS-NPs, PSar-NPs, PLL-PSar-NPs, CS-PSar-NPs were determined by the photon correlation spectroscopy method. The strength of fluctuating laser light produced by colloidal solution or nanoparticles can be measured by the Delsa Nano C instrument in the presence of an electric field. The nanoparticles were mixed with purified water during the measurement (1:10), and 0.7 mL of the sample was put in the electrophoretic flow cell. The triplicate study was conducted at 25 °C at an angle of 90°.

### 4.2. Zeta potential

When measuring zeta potential, the DelsaNano C detects the scattered light from the particles by combining incident light (reference light) with the scattered light. The amount of frequency shift *V*_d_ of scattered light is related to the mobility of particles (U) (Equation (1)):

(1) $$Vd=\frac{Uq\;}{2\pi }\;\;\text{cos}\;\frac{ϴ}{2}=\frac{Un\;}{\lambda }\;\text{sin}ϴ$$

where *q* is the scattering vector, ***λ*** is the wavelength of the incident light, *n* is the refractive index of a medium, and *ϴ* is the scattering angle. In many aqueous solutions containing an electrolyte and nanoparticles, zeta potential can be calculated from the Smoluchowski equation (Equation (2)):

(2) $$\mathrm{Zeta}\;\mathrm{potential}\;(Z)=\frac{\eta }{\epsilon ϶}U$$

where ***ϵ*** and ***϶*** are dielectric constants in a vacuum and of the solvent, respectively.

The 0.7 mL sample, which was previously diluted with distilled water, was placed in the electrophoretic flow cell during the zeta potential calculation and measured at a dispersion angle of 120° and 25 °C. The calculation was taken in triplicates for 80 cycles.

### 4.3. Scanning electron microscopy (SEM) & transmission electron microscopy (TEM)

The scanning electron microscopy of the prepared nanoparticles was performed by JSM-IT800 Field Emission Scanning Electron Microscope (Tokyo, Japan). Samples are fixed and sputtered on metal plates with a gold–palladium combination at a thickness of 100 Å and observed at an elevated voltage of 20 kV. To determine the morphology of the CS-PSar-NPs-EGFRvIII formulations and for proper magnificent images, TEM was performed (Hitachi 7500, Tokyo, Japan). Before conducting TEM, the nanoparticles were coated with carbon and put in stained copper grid phosphotungstic acid (1%). The digital micrograph-generated software interprets developed images from TEM studies.

### 4.4. Atomic force microscope (AFM)

Studies of AFM (Demchenkov et al., 2021) were conducted under special conditions in Hitachi AFM5300E, such as high vacuum and semi-contact 23. Samples can be run in AFM studies from −120 °C to 800 °C. In two dimensions, i.e. length and width wise, the AFM prob moves. In the microscopic sender slide, one drop of the nanosuspension of various nanoparticles was placed, and the slide was kept to dry at room temperature. The 3D and 2D images were created, and average parameters of roughness and kurtosis were calculated accordingly using Nova Px Control software, Moscow.

### 4.5. DSC analysis

It is important to understand the relationship between GTB and different polymers, i.e. chitosan (CS), polysarcosin (PSar), and PLL, and their nanoparticular drug-loaded composites, i.e. CS-NPs, PSar-NPs, PLL-PSar-NPs, and CS-PSar-NPs. The thermal behavior was investigated with SHIMADZU DSC-60 Plus Series Differential Scanning Calorimetry, Tokyo, Japan. During the experimental run, the temperature range was set in the inert nitrogen atmosphere at 25–300 °C. In a hermetically sealed aluminum pan, approximately 4 mg of samples were taken at 15 °C/min. The Origin Pro 8.5 software helps to plot and interpret endothermic plots (Anjum et al., 2019).

### 4.6. Fourier transform infrared spectroscopy (FT-IR)

To better understand the conjugation of GTB and EGFRvIII in polymeric nanoparticle surfaces and to crosscheck proper covalent linkage within polymeric nanoparticles, Fourier Transform Infrared Spectroscopy was used. By using the traditional KBr disk/pellet method, the drug (GTB) and lyophilized polymeric-conjugated nanoparticles (CS-NPs-EGFRvIII) were obtained (Model IRTracer-100; Shimadzu, Tokyo, Japan). Before using a hydraulic compactor to make uniform pellets from crushed KBr and sample (5-FU & lyophilized CS-NPs-EGFRvIII), the 1:100 ratio of crushed KBr and sample (GTB, lyophilized CS-NPs, lyophilized CS-NPs-EGFRvIII) was taken. For all scans, FT-IR measurements were made from 4000 to 400 cm^−1^ with a resolution of 5 cm^−1^.

### 4.7. Encapsulation efficiency of nanoparticles

The nanoparticles were first centrifuged at 15,000 rpm for 1 h. The resultant supernatant was considered for HPLC analysis to find the amount of drug encapsulated within nanoparticles. The encapsulation efficacy was calculated using the following formula:

$$\text{Drug}\;\text{entrapment}\;\text{efficacy}\;(\%)\;=\;\frac{\mathrm{Total}\text{ GTB hydrocholoride}-\;\mathrm{Free}\text{ GTB hydrocholoride }}{\text{Total GTB}}\times 100$$

The high-pressure liquid chromatographic instrument (Shimadzu HPLC model: LC-2050 series) was used for HPLC determination. For this analysis, the Luna C-18 column (250 mm, 4.6 mm; 5 μl) was considered. Up to 20 μL of injection, volume was maintained, and the SPD 20A UV–visible detector was used at 275 nm. Data acquisition has been accomplished by the use of LC-Solution tools. The 10:90 acetonitrile: water ratio was considered as the mobile phase when orthophosphoric acid and triethylamine were used to change the pH to 7.2. Using 0.45 μ Millipore membrane filters, the mobile phase was well filtered. The HPLC method was validated analytically for the assay of GTB hydrochloride (*r*^2^:0.9976).

### 4.8. *In vitro* GTB release from nanoparticles

For *in vitro* dialysis studies of GTB and drug-loaded various polymer nanoparticles, dialysis membrane (Spectra Por S/P 2 Dialysis Membrane, 12,000–14,000 Dalton 25 mm) was used. The membrane was soaked into pH 6.8 buffer solution overnight. The socked dialysis begs were filed separately with drugs and various nanoparticles (equivalent to 5 mg GTB), and the begs were tied to nylon thread dialysis tubes. The dialysis tubes were suspended in a 250 mL pH 6.8 phosphate buffer solution, maintaining the temperature at 37 ± 2 °C and sustaining Teflon Magnetic Stirrer Mixer Stirring Bar Rod Bead speed at 50 rpm. An aliquot number of samples were removed, and volume was made with fresh phosphate buffer solution (PBS). The experiment was conducted thrice, and the quantity withdrawn was filtered using a 0.22 μm membrane filter before running the experiment in the previously described HPLC method. The cumulative percentage of drug release results was articulated in average (percent) ± standard deviation. From various kinetic studies, it was revealed that drug release follows zero-order kinetics from polymeric nanoparticles.

### 4.9. Preparation of fluorescent nanoparticles

The fluorescent nanoparticles were used to emphasize the process in the culture medium. The fluorescent nanoparticles have a 700–750 nm excitation wavelength and an emission of 750–800 nm. In this experiment, rhodamine 123 and Nile Red were used as our fluorescent markers in this experiment (Machado et al., 2021). The Nile Red is lipophilic and insoluble in water, mimicking nanoparticles' characteristics. Whereas rhodamine 123 imitates the behavior of GTB, it shows the same characteristics in terms of solubility. Rhodamine 123 and Nile Red-loaded polymeric nanoparticles were prepared as per the procedure mentioned above, except for adding GTB hydrochloride in nanosuspension. Ricinoleic acid triglyceride oil was used to prepare Nile Red control solution, and rhodamine control solution was prepared using HPLC-grade water.

### 4.10. Preparation of EGFRvIII-conjugated polymeric nanoparticles

The chemical conjugation method was implemented to immobilize EGFRvIII. Thiolated EGFRvIII was first primed for 45 min at room temperature by mixing EGFRvIII with 2-iminothiolane. Individually CS-NPs, PSar-NPs, PLL-PSar-NPs, CS-PSar-NPs, and 1,2-dioleoyl-sn-glycero-3-phosphoethanolamine-*N*-[4-(*p*-maleimidophenyl) butyramide] [MPB-PE] (Piao et al., 2021) was applied progressively to the surface thiolated EGFRvIII and incubated overnight in an inert nitrogen environment at 4 °C to start the coupling reaction between prepared nanoparticles. While preparing, the stirring speed was kept at 200 rpm. All four polymeric nanoparticles conjugated by EGFRvIII (CS-NPs-EGFRvIII, PSar-NPs-EGFRvIII, PLL-PSar-NPs-EGFRvIII, CS-PSar-NPs-EGFRvIII) were purified by centrifugation for 45 min at 18,000×*g*. The pallets obtained were collected and suspended in a phosphate buffer solution of pH 6.8 and washed three times. Bio-Rad protein kit-1 (Quick StartTM-5000201EDU, Bio-Rad, Hercules, CA) was quantified for EGFRvIII. By subtracting the approximate amount of unbound EGFRvIII from the initial amount which was incorporated into the polymeric suspension, the amount of EGFRvIII conjugated on the surface of different polymeric nanoparticles can be defined.

### 4.11. Cell culture

The human ovarian tumor cell lines, i.e. IGR-OV1, OVCAR-5, and OVCAR-8 were provided by the National Center, Pune, India. In the presence of 15% fetal bovine serum (FBS), the cells were cultured in RPMI-1640 and McCoy's 5A medium. In order to avoid contamination and maintain selective pressure on transfected DNA, prophylactic streptomycin (120 μg/mL) and penicillin (100 units/mL) was introduced into the culture medium. Rest all the reagents utilized for this experiment were purchased from Sigma-Aldrich, Bangalore, India.

### 4.12. Cellular uptake of polymeric nanoparticles

In this experiment, polymeric nanoparticles were strained with tracer dye (1:500 *v/v* dilution) in the presence of pH 7.4 phosphate buffer. The OVCAR-8 cells were seeded into 2.0 × 103/50 µL per well in 96-well plates for 12 h. The cells were continuously incubated for 24 h at 37 °C, maintaining 5% CO_2_ flow while treated with CS-NPs-EGFRvIII, PSar-NPs-EGFRvIII, PLL-PSar-NPs-EGFRvIII, and CS-PSar-NPs-EGFRvIII. During incubation, cells were periodically washed at 2nd, 4th, and 24th hours using phosphate buffer (PBS) solution. Further, the cells were fixed with 4% formaldehyde solution for 20 min at 25 °C. The fixed cells were washed with phosphate buffer solution, and then dropwise 0.5 µg/mL Hoechst dye was added and further incubated for 20 min at room temperature. After incubation, once again, cells were washed with phosphate buffer solution and under STELLARIS Confocal Microscope (Leica Microsystem, Grove, IL, USA). Under 60× cells were observed to identify the internalization of polymeric nanoparticles.

### 4.13. Specificity of drug and EGFRvIII-conjugated polymeric nanoparticles

To confirm the specific binding of EGFRvIII-conjugated polymeric nanoparticles onto the OVCAR-8 cells, a flow chamber assay was performed. Further, the ratio of targeted nanoparticles binding and non-targeted nanoparticles bindings was calculated. To enhance the specificity of EGFRvIII-conjugated polymeric nanoparticles, VEGF secreting cells were used as a control. The binding ratio of EGFRvIII-conjugated nanoparticles (CS-NPs-EGFRvIII, PSar-NPs-EGFRvIII, PLL-PSar-NPs-EGFRvIII, CS-PSar-NPs-EGFRvIII) was two-fold higher than the fibroblast cells. These results indicate the use of anti-VEGF molecules (CS-NPs-EGFRvIII, PSar-NPs-, EGFRvIII PLL-PSar-NPs- EGFRvIII, CS-PSar-NPs-EGFRvIII) can significantly increase the delivery of nanocomposite to overlain cancer cells.

### 4.14. Targeting efficiency test (*in vitro*)

The vascular endothelial growth factor (VEGF) was targeted by its antibody EGFRvIII, which ultimately results in the inhibition of R-terminal binding of VEGF (Kaufman et al., 2021). By using a flow chamber system and under dynamic conditions, the specific binding of EGFRvIII into extracellular VEGF was determined. The flow chamber system is composed of the fluorescent microscope, syringe pump, and finally, a microfluid chamber. The microfluid chamber comprised of a rubber gasket, deck, and cells for six well plates. The entire chamber was exposed to a fluorescence microscope. Further, the Olympus DP controller management program observed the fixation between the cells and the particles. The OVCAR-8 cells, fibroblast, VEGF were selectively seeded into six-well plates and cultured overnight (1 × 10^3^ cells/well). The EGFRvIII-conjugated polymeric nanoparticles were mobilized into the system and passed through the stationary cells. By the VEGF screening, the interaction and attachment between polymeric nanoparticles and targeted cells were determined. The fixation of EGFRvIII-conjugated polymeric nanoparticles was observed under a fluorescence microscope, and hence, the number of polymeric nanoparticles were counted, which was adhered to the cell surface and calculated the ratio of targeted and non-targeted nanoparticles. The number of adhered polymeric nanoparticles was calculated and counted under a fluorescence microscope (Olympus IX71, Tokyo, Japan).

### 4.15. MTT cytotoxicity assay

The anticancer activity of EGFRvIII-conjugated GTB-loaded cationic nanoformulations, i.e. CS-NPs-EGFRvIII, PSar-NPs-EGFRvIII, PLL-PSar-NPs-EGFRvIII, CS-PSar-NPs-EGFRvIII, unconjugated nanoparticles, and free GTB, were determined against OVCAR-8. To evaluate the toxicity of EGFRvIII-conjugated nanoparticles, OVCAR-8 cells were seeded into 96-well tissue culture plates at a concentration of 2.0 × 10^3^/50 µL per well of complete 15% fetal bovine serum medium for 12 h in an incubator. The cells were further treated with EGFRvIII-conjugated nanoparticles consisting of 0.25, 0.5, 0.75, and 1.00 µg/mL of GTB. After 24 h, 15 µL of 3-(4,5-dimethylthiazol-2-yl)-2,5-diphenyl tetrazolium bromide (MTT) was added into the 96-well tissue culture plates and incubated for 4 h. After incubation, the medium was removed. The formed formazan crystals were dissolved in 100 µL of lysis buffer (pH 4.7) and 80 µL DMSO & 12% of SDS. The absorbance of the solution was determined at 570 nm using BioTek™ LED Cubes for Imaging Multi-Mode Readers (Fisher Scientific, Waltham, MA).

## 5. Result and discussion

In this experiment for the treatment of ovarian cancer, GTB-loaded cationic polymeric nanoparticles were prepared for the intravesical drug delivery. Four different polymeric nanoparticles, namely, chitosan nanoparticles (CS-NPs), polysarcosine nanoparticles (PSar-NPs), chitosan & polysarcosine-fabricated nanoparticles (CS-PSar-NPs), and poly-l-lysine & polysarcosine fabricated nanoparticles (PLL-PSar-NPs) were prepared and evaluated. The prepared polymeric nanoparticles were assessed to ensure desired particle size, cationic zeta potential, surface morphology, control release profiling, cellular internalization and EGFRvIII conjugation effects, and anticancer efficacy of nanoparticles on various ovarian cell lines, i.e. IGR-OV1, OVCAR-5, and OVCAR-8.

All the polymers like chitosan (CS), polysarcosine (PSar), poly-l-lysine (PLL), and their conjugates were used for their previously reported excellent biocompatible properties and excellent mucoadhesive drug delivery properties.

The mean particle size and polydispersity index (PDI) of uncoated nanoparticles (CS-NPs and PSar-NPs) was found to be within the range of 110.25–145.5 nm and 0.15–0.25. However, after coating with different polymers, the particle size of the coated nanoparticles (CS-PSar-NPs & PLL-PSar-NPs) increased in a range of 174.53–198.34 nm with non-significant changes in PDI. It was observed more specifically that coating of hydrophilic polymers (chitosan, polysarcosine & poly-l-lysine) significantly increases the particle size of the nanoparticles, as can be seen in Table 1.

**Table 1.** Particles size distribution and Zeta potential values of coated and uncoated nanoparticles.

Polymeric nanoparticle formulations	Mean diameter (nm) (±SD)	Polydispersibility index	Zeta potential (mV)
CS-NPs	145.5 ± 6.54	0.25	35.56 ± 4.67
PSar-NPs	110.25 ± 8.45	0.15	30.45 ± 5.46
PLL-PSar-NPs	174.53 ± 7.22	0.21	37.22 ± 6.61
CS-PSar-NPs	198.34 ± 8.22	0.26	34.41 ± 4.26

In PSar-NPs, the smaller particle size was obtained; however, all the particles were within the 200 nm below range; hence, reticuloendothelial opsonization can be avoided. In addition, it may also be possible to increase nanoparticles' permeation in various ovarian cell epithelial cancer tissues. During the experiment, it was observed that the presence of poly-l-lysine (PLL) in the nanoparticle's solution enhances the particles' size and cationic zeta potential. Moreover, chitosan (CS), polysarcosine (PSar) & poly-l-lysine (PLL) showing cationic nature; therefore, prepared nanoparticles were found to have colloidal nature with limited agglomeration. In Calvo et al. (1997b) article on novel hydrophilic chitosan‐polyethylene oxide nanoparticles, similar results were witnessed.

Bhattamisra et al. (2020) used cell-based experimental and pharmacodynamic models to develop rotigotine-loaded chitosan nanoparticles (RNPs) for nose-to-brain delivery. The zeta potential of the RNPs was found to be cationic (+25.53 ± 0.45 mV) due to the presence of amino groups on the chitosan molecule that were not neutralized by TPP. Furthermore, the nanoparticles were less than 200 nm in size, suggesting that this formulation could be ideal for drug delivery from the nose to the brain.

In our current research, when GTB were coated with hydrophilic polymers (chitosan, polysarcosine & poly-l-lysine), the zeta potential of the nanoparticles were significantly increased, and moreover, the particle size was found to be less than 200 nm, which could validate our research findings with Bhattamisra et al. (2020) work.

SEM photomicrography study was considered to understand the morphological differences between blank and drug-loaded nanoparticles (Figure 1(A–H)). All the cationic nanoparticles show smooth regular surfaces with spherical texture. During SEM analysis, it was witnessed, the polymeric nanoparticles are showing somewhat shrink in the presence of electrical bombardment, which might be due to the thermal fluctuation, which causes evaporation of liquids from the internal portion of the nanoparticles. The PLL-PSar-NPs and CS-PSar-NPs show larger particle sizes in SEM analysis; this might be due to the encapsulation of nanoparticles with two polymers.

**Figure 2.**
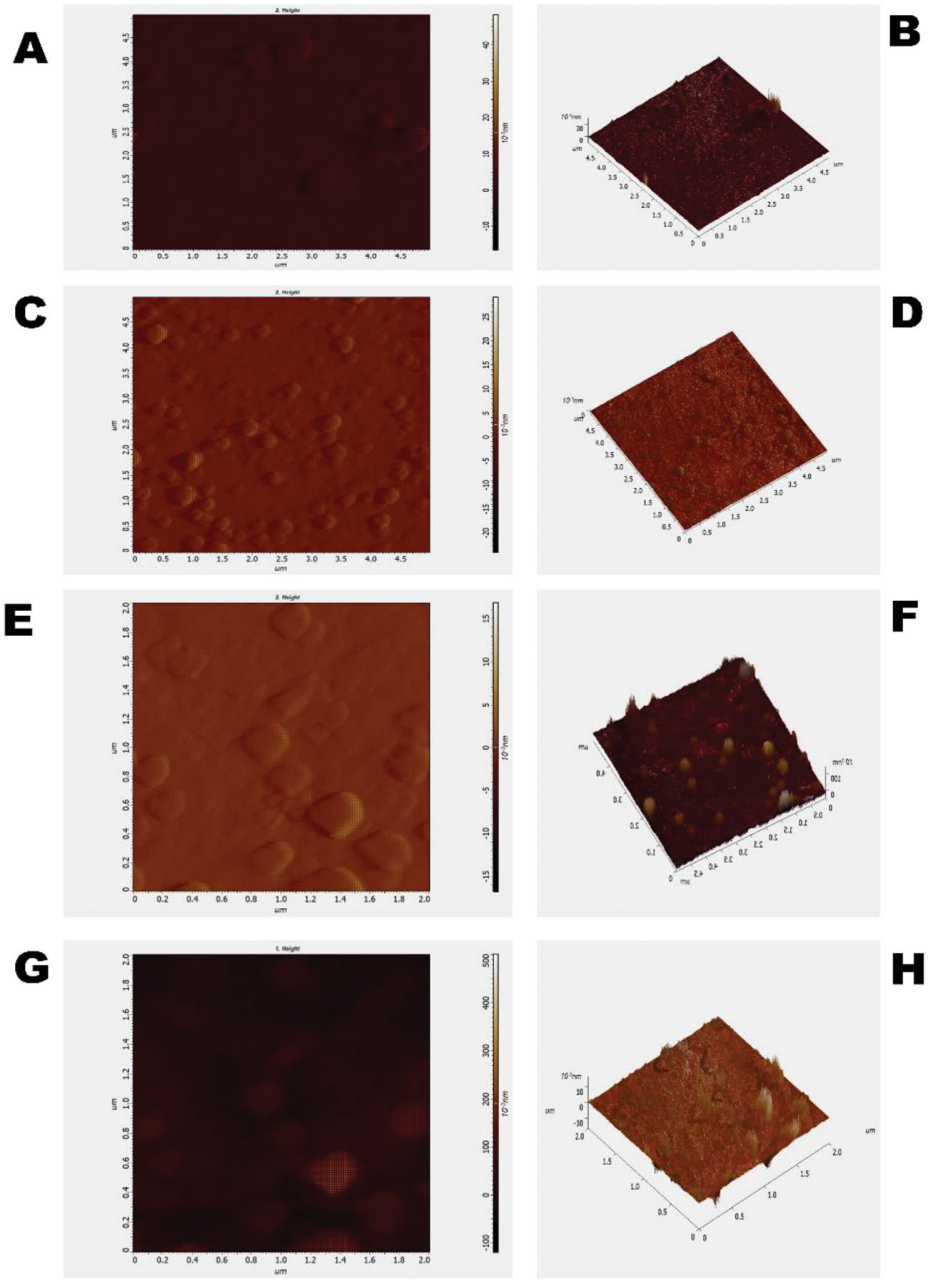
(A) 2-dimentional AFM batch of polymeric gemcitabine-loaded CS-NPs, (B) 3-dimentional AFM of polymeric gemcitabine-loaded CS-NPs, (C) 2-dimentional AFM batch of polymeric gemcitabine-loaded PSar-NPs, (D) 3-dimentional AFM of polymeric gemcitabine-loaded PSar-NPs, (E) 2-dimentional AFM batch of polymeric gemcitabine-loaded PLL-PSar-NPs, (F) 3-dimentional AFM of polymeric gemcitabine-loaded PLL-PSar-NPs, (G) 2-dimentional AFM batch of polymeric gemcitabine-loaded CS-PSar-NPs, and (H) 3-dimentional AFM of polymeric gemcitabine-loaded CS-PSar-NPs.

The atomic force microscopy (AFM) or scanning force microscopy experiment was performed in high vacuumed condition and semi-contact mode. AFM was carried out to characterize the surface morphology of CS-NPs, PSar-NPs, PLL-PSar-NPs, & CS-PSar-NPs. As per (Figure 2(A–H)), most particles of the prepared polymeric nanoparticles have a rough surface, and particle size shows around 100–170 nm, which coincided with particle size data obtained from Delsa Nano C. On the other hand, GTB-loaded PLL-PSar-NPs, CS-PSar-NPs showing smooth surface with significantly larger nanoparticle size; indicating conjugation of two polymers on the surface of the nanoparticles. AFM helps to determine the nanomaterials skewness, roughness, kurtosis. The particles' 3D images of AFM studies indicate a good correlation between the particle size measured by Delsa Nano C photon correlation spectroscopy (PCS). The skewness, roughness, and kurtosis were reported as 0.290, 3.013 nm, and 0.645, respectively, indicating the spherical and symmetrical nature of all the polymeric nanoparticles' surfaces.

With CS-PSar-NPs formulations, the highest drug encapsulation efficacy can be obtained, which is having 76% loading of GTB. Whereases, uncoated PSar-NPs shows poor loading capacity; only 24%, which is due to the higher water solubility profile of PSar and PSar coating with CS, however, increases the higher-fold of the nanoparticles' loading capacity, which might be due to the coating of PSar over CS, which is having a strong affinity with GTB. Moreover, PLL had lower hydrophilicity, which means lower affinity with GTB and hence lower drug loading capacity recorded. The encapsulation efficacy (%) profile of GTB in various nanoparticles prepared for this study (CS-NPs, PSar-NPs, PLL-PSar-NPs, and CS-PSar-NPs) is presented in Table 2.

**Table 2.** The encapsulation efficacy (%) of CS-NPs, PSar-NPs, PLL-PSar-NPs & CS-PSar-NPs (*n* = 3).

Polymeric nanoparticles formulation	Encapsulation efficacy (%)
CS-NPs	42.6 ± 4.78
PSar-NPs	24.00 ± 0.23
PLL-PSar-NPs	45.89 ± 1.83
CS-PSar-NPs	76.00 ± 4.28

**Figure 3.**
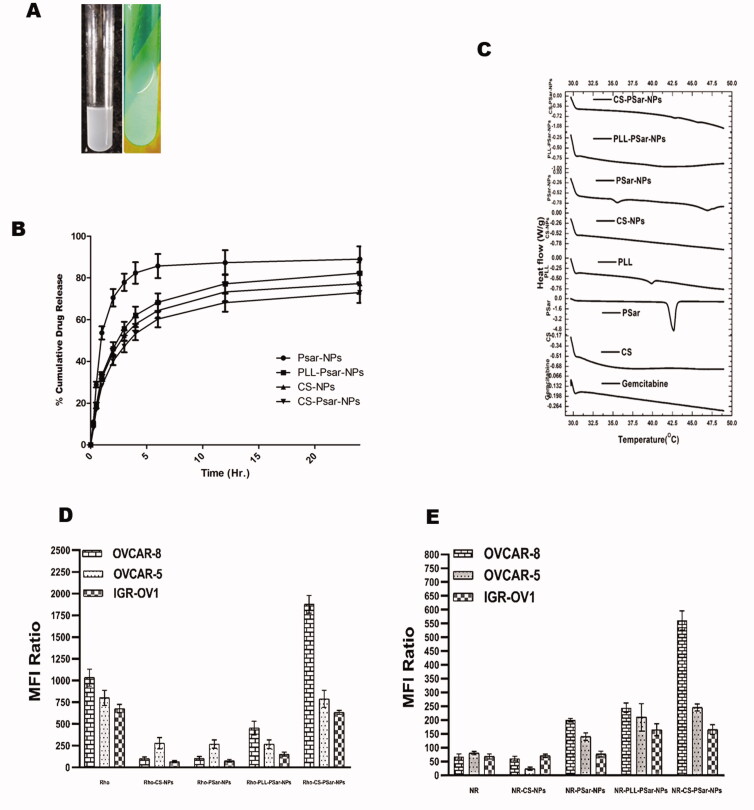
(A) freshly prepared polymeric nanoparticles in normal condition and polymeric nanoparticles in UV light; scattering was witnessed. (B) *In vitro* release profile of Gemcitabine from cationic nanoparticles formulation in pH 6.8 buffer solution (*n* = 3 ± SD). (C) DSC thermograms gemcitabine and nanoparticles excipients and prepared nanoparticles. The uptake of rhodamine 123 in the upper panel (D) and lower panel; (E) Nile red-loaded nanoparticles in OVCAR-8, OVCAR-5, and IGR-OV1 cells. With the help of a flow cytometer, the fluorescence intensity (MFI) was analyzed. Cellular uptake of fluorescent dyes was calculated with the mean fluorescence intensity (MFI) ratio of the corresponding formulation vs. autofluorescence of untreated cells.

Scattering was observed between freshly prepared polymeric nanoparticles in standard conditions and polymeric nanoparticles exposed to UV light (Figure 3(A)), indicating the formation of colloidal nanoparticles solution. The *in vitro* drug release profiling of the cationic polymeric nanoparticles is observed in Figure 3(B). Almost 72.99 ± 4.97% of drug releases were observed in CS-PSar-NPs loaded with GTB hydrochloride in 24 h. PSar-NPs loaded with GTB, however, show 88.97 ± 6.18% drug release, and PLL-PSar-NPs loaded with GTB hydrochloride show 82.29 ± 5.19% drug release. Similarly, CS-NPs indicate a 77.27 ± 4.89% release of drugs. During the process of GTB encapsulation within polymers, it was observed that drugs remain adsorbed onto the outer surface. Therefore, the initial burst effects and subsequent plateau can be seen. In this experiment, to maintain sink condition, dialysis tubing was utilized. However, these data need to be compared with cellular uptake and cytotoxic data.

Figure 3(C) explains about DSC thermograph of GTB. The graph explains about different thermal behavior of GTB with different polymers and the prepared composition of nanoparticles. All the polymeric nanoparticles of GTB hydrochloride suggest that there is no possible interaction with the drug with polymers. As such, no significant endothermic changes were observed with drugs and polymers in the prepared polymeric nanoparticles. However, PSar endothermic peak at 42.5 °C has shifted to 47.5 °C in GTB-loaded PSar-NPs. This might be the presence of Pluronic^®^ F68, a nonionic surfactant, within the PSar-NPs. It was noteworthy that CS-PSar-NPs have not shown such type of interaction even it has Pluronic^®^ F68 within its preparation. In *in vitro* drug release studies, it was also observed that due to the presence of Pluronic^®^ F68 within CS-PSar-NPs, the release of GTB from the nanoparticles is much higher as compared to the other drug-loaded nanoparticles, which indicates Pluronic^®^ F68 strongly interacts with CS and PSar. This interaction helps in strong and steady drug release from the nanoparticles. Nevertheless, the interaction of GTB with nanoparticles surfaces was found to be the key parameter that can affect drug release. The GTB has also changed the thermal behavior of PLL-PSar-NPs, whereas no significant endothermic peak was missing.

**Figure 4.**
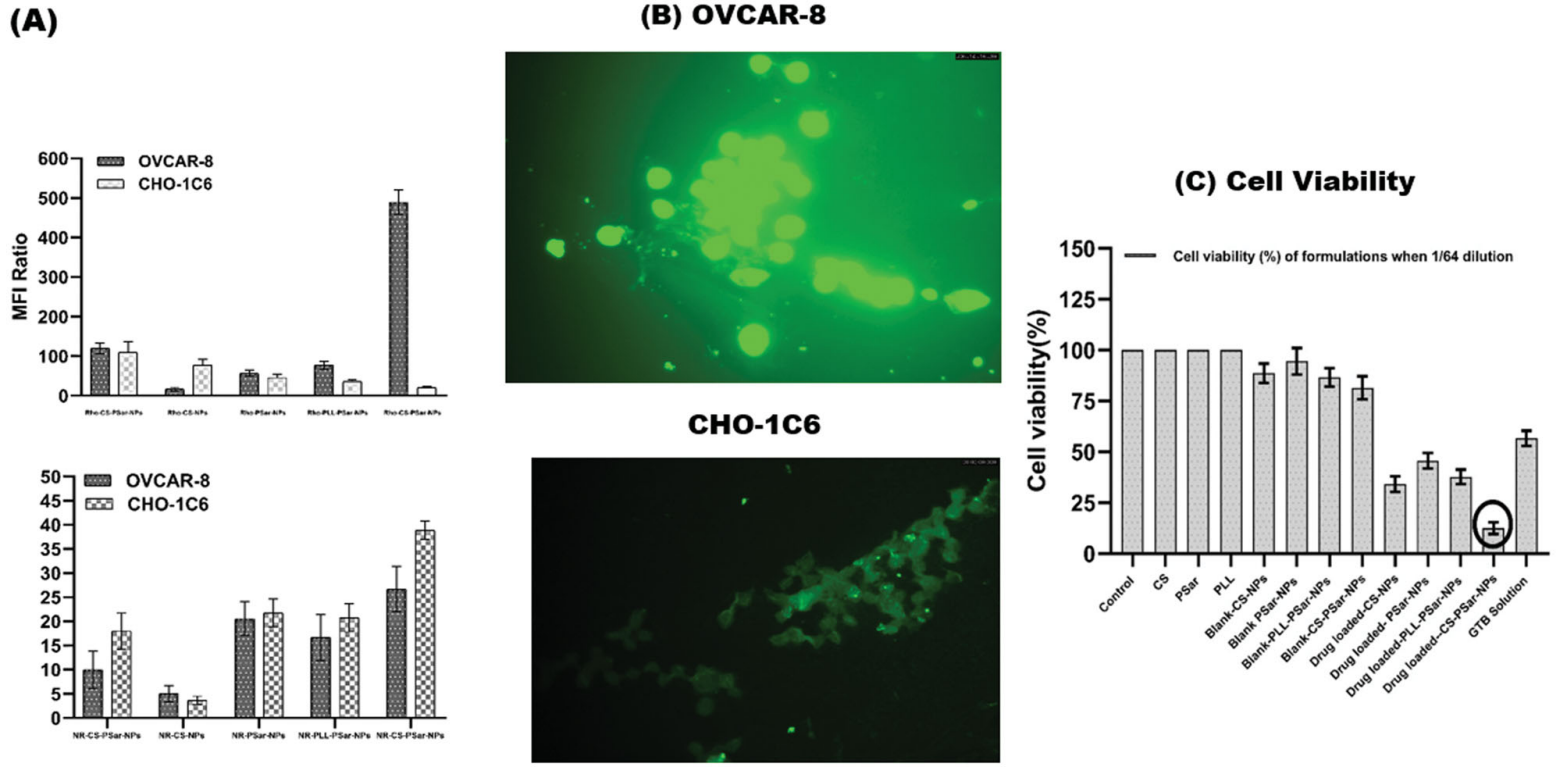
The uptake of nanoparticles in normal ovarian cell line (CHO-1C6) and in ovarian cancer cell lines (OVCAR-8) (A). Mean fluorescence intensity (MFI) value was obtained by flowcytometric analysis of ovarian cancer cell lines (OVCAR-8) and normal ovarian cell lines (CHO-1C6)treated with rhodamine 123 and Nile Red-loaded polymeric nanoparticles. Cellular uptake of fluorescent dyes was calculated with the mean fluorescence intensity (MFI) ratio of the corresponding formulation versus autofluorescence of untreated cells. Photomicrographs of the OVCAR-8 and CHO-1C6 cells treated with Rho-CS-PSar-NPs nanoparticles (×1000) (B). Cell viability (%) of different formulation with drug and without drug loading against OVCAR-8 cell line (C).

The GTB is reported to have degradation in an acidic environment (Jansen et al., 2000). Therefore, intravehicular administration of GTB would be challenging and could deplete drug loading; hence, loss of therapeutic dosing can be possible. This phenomenon could also affect the release of drugs in the presence of pH 6.0 phosphate buffer solution, which results in lower cumulative drug release of GTB. As per Sangram De & Robinson (2003) & Samal et al. (2012), research and review finings, chitosan (CS), and poly-l-lysine–alginate (PLL) presence in nanoparticles could enhance the net cationic or positive charge of the surface of the nanoparticles, which could sometimes increase particles size of the nanoparticles as well. As per Calvo et al. (1997b) research findings, the presence of PLL and CS within the formulation increase the particle size but PLL would not be having any significant effect on drug penetration but CS has (Calvo et al., 1997a). As per De and Robinson's (2003) studies (Robinson et al., 2003), it was recognized that the release of drugs from PLL and CS coated nanoparticle's surface significantly depends on the concentration of sodium chloride present in the dissolution medium. As per Hejjaji (2018), chitosan nanoparticles have excellent mucoadhesive properties when administered with tripolyphosphate. Therefore, chitosan and polysarcosine combination could enhance the mucoadhesive property and intravesical drug delivery system. CS-PSar-NPs were found to have excellent uptake in IGR-OV1, OVCAR-5, and OVCAR-8 cell lines in the presence of hydrophilic rhodamine 123 and hydrophilic Nile red marker. In OVCAR-8, OVCAR-5, and IGR-OV1 cells, the uptake of rhodamine 123 in the upper panel (D) and lower panel (E) Nile red loaded nanoparticles is shown in Figure 3(D,E). The fluorescence intensity (MFI) was measured using a flow cytometer. The mean fluorescence intensity (MFI) ratio of the corresponding formulation vs. autofluorescence of untreated cells was used to calculate cellular uptake of fluorescent dyes. With the exposure to a different ranges of cell lines, the formulations were tested. The fluorescence intensity of the OVCAR-8 cell line treated with Rho-CS-PSar-NPs shows higher MFI than the standard CHO-1C6 cell lines. It was also observed that CHO-1C6 cell lines have higher uptake in Nile Red dye. It was understood that hydrophobic drug delivery would be a limiting step of this research work. The selective delivery of Rho-CS-PSar-NPs can be the best implication for ovarian cancer target therapy (Figure 4(A)). Photomicrographs of the OVCAR-8 and CHO-1C6 cells treated with Rho-CS-PSar-NPs nanoparticles were measured in 1000× magnification and reported in Figure 4(B). The cell viability (%) was calculated for GTB loaded different polymeric nanoparticles against the OVCAR-8 cell line (Figure 4(C)). The blank CS, PSar, and PLL show non-toxicity and GTB-loaded CS-NPs PSar-NPs, PLL-PSar-NPs, and CS-PSar-NPs show higher cytotoxicity as compared with simple GTB solution when treated in OVCAR-8 cell lines. Noticeably, GTB-loaded CS-PSar-NPs show higher toxicity against normal GTB solution. For the intravesical chemotherapy, GTB-loaded CS-PSar-NPs seem to be the best formulation for potential anticancer effects against ovarian cancer cells.

**Figure 5.**
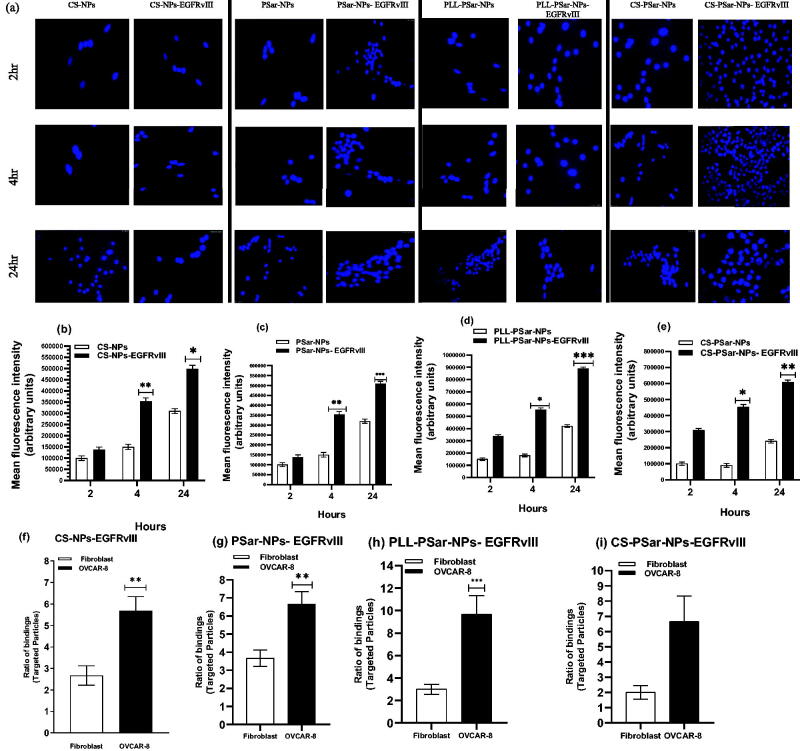
Cellular uptake and specificity of EGFRvIII-conjugated nanoparticles when treated in OVCAR-8 cell lines with Dil-leveled nanoparticles; CS-NPs, CS-NPs-EGFRvIII, PSar-NPs, PSar-NPs- EGFRvIII, PLL-PSar-NPs, PLL-PSar-NPs- EGFRvIII, CS-PSar-NPs, CS-PSar-NPs-EGFRvIII and observed under confocal microscope at 2, 4, and 24 h incubation (a). Quantitative analysis of cell internalization showed signal intensity compared between CS-NPs, CS-NPs-EGFRvIII, PSar-NPs, PSar-NPs- EGFRvIII, PLL-PSar-NPs, PLL-PSar-NPs- EGFRvIII, CS-PSar-NPs, CS-PSar-NPs-EGFRvIII (b–e). The specific binding capacity of CS-NPs-EGFRvIII, PSar-NPs- EGFRvIII, PLL-PSar-NPs- EGFRvIII, CS-PSar-NPs-EGFRvIII was analyzed in fibroblast (low VEGF secreting cells) and OVCAR-8 secreting cells. Cells were treated with the nanoparticles and their binding efficiency was analyzed using a flow chamber assay (f–i). Data are presented as mean ± SD. The graph represents data from two independent experiments. *p* < .05, Student *t*-test.

The biological nature of cancer cells in the outer layer can be used as a target for cancer treatment. Overexpressed vascular endothelial growth factor (VEGF) and its receptor are frequently found in the leaky vasculature of cancer cells. VEGF has been found in cervical and breast cancers, according to several studies. Anti-VEGF antibodies, such as epidermal growth factor receptor variant III (EGFR vIII), could be used to target these VEGF. To learn more about target specificity, polymeric nanoparticles, such as CS-NPs, PSar-NPs, PLL-PSar-NPs, and CS-PSar-NPs, were scaffolded with EGFR vIII then drugged using an active targeting approach. To demonstrate the importance of targeting the upshot of EGFR vIII scaffold nanoparticles, namely, CS-NPs-EGFRvIII, PSar-NPs-EGFRvIII, PLL-PSar-NPs-EGFRvIII, and CS-PSar-NPs-EGFRvIII, the OVCAR-8 cell lines were incubated with non-targeted nanoparticles, i.e. CS-NPs, PSar-NPs, PLL-PSar-NPs. Confocal microscopy was used to examine the comparative profiling of specific internalization and cellular distribution (Figure 5(a)). The fluorescence intensity of particles uptake was measured and reported in Figure 5(b–e). To identify the significance of targeting and internalization effects of CS-NPs-EGFRvIII, PSar-NPs-EGFRvIII, PLL-PSar-NPs-EGFRvIII, CS-PSar-NPs-EGFRvIII against the non-targeted polymeric nanoparticles under inert conditions for 2, 4, and 24 h, the distributed cells were analyzed using confocal microscopy. By confocal microscopy, both targeted and non-targeted nanoparticles signals were identified in OVCAR-8 cells.

Moreover, quantitative signal intensities of particle uptake were also analyzed. The confocal images help to identify the signals of targeted and non-targeted nanoparticles in the OVCAR-8 cells. The obtained signals were visualized at 4 h and 24 h after incubation. Due to the EGFRvIII target specificity, EGFRvIII-conjugated nanoparticles show higher affinity than the non-targeted nanoparticles after an initial 2 h of incubation. At 24th hours after incubation, quantitative studies significantly revealed that all the EGFRvIII-conjugated nanoparticles have almost two-fold internalization capacity than that of unconjugated nanoparticles with time-dependent cell entry. Therefore, harboring drug and EGFRvIII-conjugated polymeric nanoparticles would have a higher ability to enter the ovarian cancer cells after the initial second hour of incubation. After a 24-h incubation period, it was clear that EGFRvIII scaffold nanoparticles had a higher rate of internalization into OVCAR-8 cells than non-targeted nanoparticles; additionally, time-dependent cell entry was also observed.

In fibroblast (low VEGF secreting cells) and OVCAR-8 secreting cells, the specific binding capacity of CS-NPs-EGFRvIII, PSar-NPs-EGFRvIII, PLL-PSar-NPs-EGFRvIII, and CS-PSar-NPs-EGFRvIII was investigated. A flow chamber assay was used to assess the binding efficiency of cells that had been treated with nanoparticles. The specific bindings of EGFRvIII are confirmed by this flow cytometric assay. The ratio of EGFRvIII nanoparticle bindings to targeted and non-targeted nanoparticles is shown in Figure 5(b–e). The targeting specificity of each EGFRvIII scaffold nanoparticle was tested against OVCAR-8 cells, with lower VEGF-containing fibroblasts serving as a control. The binding ratio of EGFRvIII in OVCAR-8 cells is much higher than in the controlled fibroblast cells, as shown in Figure 5(f–i). According to Shein et al. (2015) (Abakumov et al., 2015), anti-VEGF nanoformulation was highly effective against VEGF + tumor cells in both *in vitro* and *in vivo* conditions, and it could also be effective against C6 glioma cells, which helps to improve target specificity and selectivity of nanoparticles. The MTT assay helps to determine the therapeutic effects of targeted nanoparticles. In the 3D culture model, the cell viability was determined. The capacity of EGFRvIII to target OVCAR-8 cells was investigated in a 3D culture model, and thus, free drug, unconjugated polymeric nanoparticles, and EGFRvIII-conjugated nanoparticles corresponding to an IC_50_ (15 mg/mL) were obtained. At initial hours, the cell viability was unchanged; whereases after 24 h, the viability of OVCAR-8 cells was significantly decreased when treated with EGFRvIII-conjugated nanoparticles, compared to uncoagulated nanoparticles. From Figure 6(a–d), it was evident that the cell viability of OVCAR-8 cells in a dose-dependent manner was significantly inhibited by EGFRvIII scaffold-target specific nanoparticles (CS-NPs-EGFRvIII, PSar-NPs-EGFRvIII, PLL-PSar-NPs-EGFRvIII, and CS-PSar-NPs-EGFRvIII).

Figure 6.(a–d) Cytotoxicity test of CS-NPs-EGFRvIII, PSar-NPs- EGFRvIII, PLL-PSar-NPs- EGFRvIII, CS-PSar-NPs-EGFRvIII against free GTB and unconjugated nanoparticles (CS-NPs, PSar-NPs, PLL-PSar-NPs, CS-PSar-NPs) against OVCAR-8 cell line when treated with different concentration of drug-loaded nanoparticles and free drug solution for 24 h. MTT assay was performed to determine the cell viability. Data are presented as mean ± SD. The graph is represents data from two independent experiments. *p* < .05, Student *t*-test. TEM images of unconjugated CS-PSar-NPs (e) and EGFRvIII-conjugated targeted CS-PSar-NPs-EGFRvIII (f). Particle size measurement of CS-NPs-EGFRvIII using Delsa Nano C instrument (g). FTIR spectroscopy of GTB, CS-PSar-NPs and CS-PSar-NPs-EGFRvIII; black shade over CS-PSar-NPs-EGFRvIII spectra indicating conjugation of EGFRvIII over CS-PSar-NPs surface (h).

**Figure:**
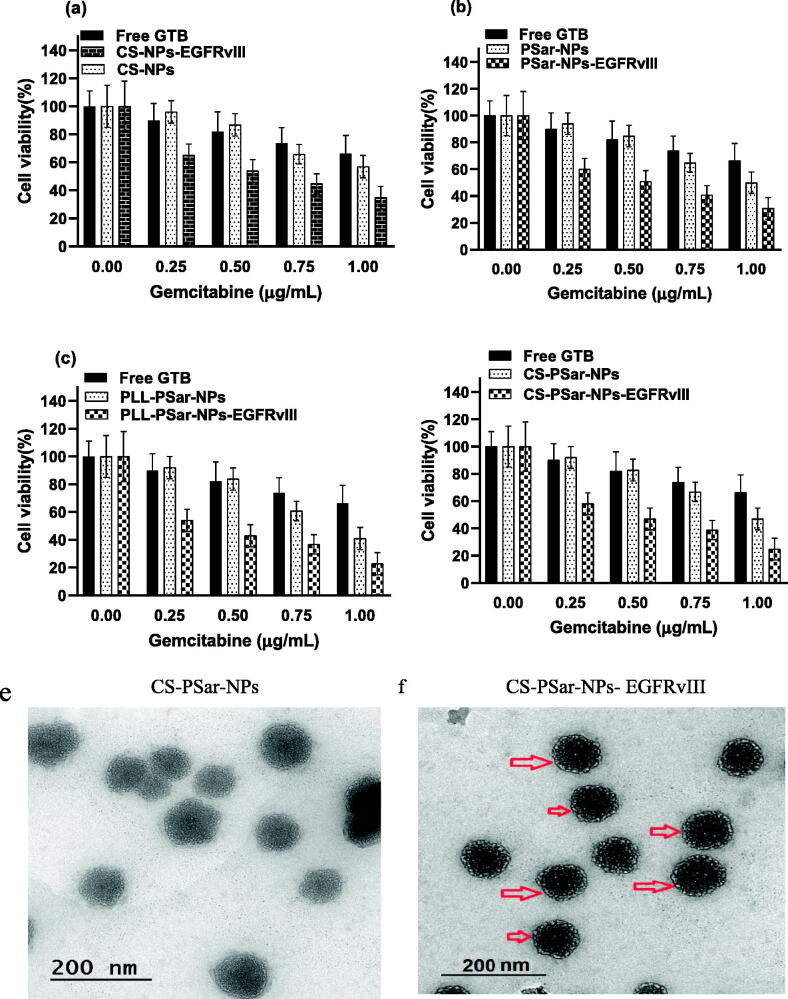

**Figure:**
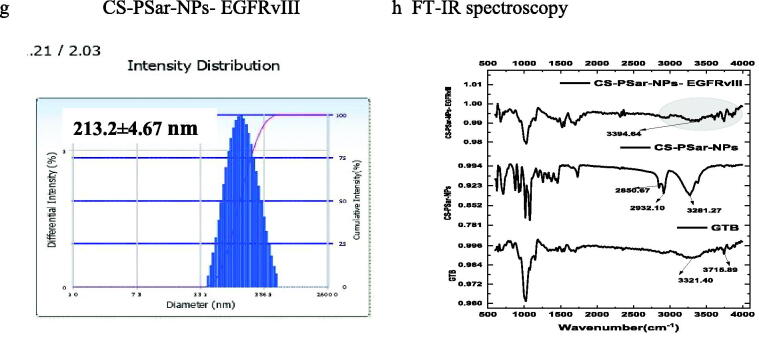

Transmission electron microscopes (TEM) for EGFRvIII-conjugated and non-conjugated nanoparticles are used to determine the morphological configuration of these formulations. The morphology of non-conjugated nanoparticles (CS-PSar-NPs) was spherical (Figure 6(e)), and particle size predicted in TEM was nearly identical to the particle size findings of the Delsa Nano C instrument (Beckman Coulter, Carlsbad, CA, USA) (Table 1). With increasing particle size, Figure 6(f) projects the morphology of the CS-PSar-NPs-EGFRvIII formulation onto the outer layer of the nanoparticles' surface, clearly suggesting EGFRvIII conjugation and projectile. As a result of the TEM study, it is possible to infer that EGFRvIII conjugation over the non-targeted CS-PSar-NPs surface was efficient, and thus, CS-PSar-NPS-EGFRvIII had become more target-specific. When measured with the Delsa Nano C instrument, the particle size of CS-NPs-EGFRvIII was found to be 213.2 ± 4.67 nm, slightly higher than that of unconjugated CS-NPs (Figure 6(g)). FT-IR spectra of the pure GTB showed the characteristic broad peak at 3321.40 cm^−1^ and 3715.89 cm^−1^, representing R_2_NH amines’ N–H stretch and amide N–H stretch. On the other hand, FTIR spectra of CS-PSar-NPS represent wide C–H stretch at 3281.27, and 2932.10 cm^−1^ represents carboxylic acid C = O stretch and alkyl C–H stretch at 2850.67 cm^−1^. Surprisingly the CS-PSar-NPs-EGFRvIII spectra show a narrow peak of amine N–H stretching at 3394.64 cm^−1^, which indicates a covalent linkage between EGFRvIII and CS-PSar-NPs, the EGFRvIII molecule successfully linked with polymer surface. Nevertheless, the less intensity of CS-PSar-NPs-EGFRvIII spectra, indicating EGFRvIII has successfully conjugated in the outer surface of the polymeric nanoparticles, but the drug has not changed its integrity (Figure 6(h)) and any chemical reactions between the drug and excipients was found to be absent. The CS-PSar-NPs-EGFRvIII spectra also indicate that the GTB and EGFRvIII had perfect amalgamation and were retained in salt form in the nanoparticle surface.

From the anticancer evaluation tests, it was evident that non-targeted (CS-PSar-NPs) and conjugated/targeted CS-PSar-NPs-EGFRvIII exhibits superior anticancer effects as compared to other EGFRvIII-conjugated nanoparticles (CS-NPs-EGFRvIII, PSar-NPs-EGFRvIII, PLL-PSar-NPs-EGFRvIII). Cell viability of OVCAR-8 cells was diminished drastically in the presence of CS-PSar-NPs-EGFRvIII as compared to free GTB solution and non-targeted GTB-loaded nanoparticles (CS-PSar-NPs). Therefore, chitosan (CS) core and polysarcosine (Psar) surface coated epidermal growth factor receptor variant III (EGFR vIII) scaffold polymeric nanoparticles (CS-PSar-NPs-EGFRvIII) seems to be a better candidate for GTB intravesical delivery for ovarian cancer.

## 6. Conclusion

Different cationic intravesical polymeric nanoparticles were prepared and characterized to effectively deliver GTB against ovarian cancer. Among GTB-loaded polymeric nanoparticles, i.e. chitosan nanoparticles, polysarcosin nanoparticles, poly-l-lysine & polysarcosin-fabricated nanoparticles, chitosan & polysarcosin-fabricated nanoparticles, it was observed that chitosan and polysarcosin-fabricated nanoparticles (CS-PSar-NPs) exhibited excellent drug loading and drug release profile with good cell internalization and anticancer effects. Combining chitosan and polysarcosin in the form of polymeric nanoparticles could be a strong choice for bio-adhesive drug delivery. Furthermore, epidermal growth factor receptor variant III (EGFR vIII) scaffolds overall polymeric nanoparticles are designed to achieve more target specificity. Again, among all molecular nanoparticles, CS-PSar-NPs-EGFRvIII shows higher cell internalization and accumulation in OVCAR-8 cells. The benefits of such EGFR vIII encroached nanoparticles could be reduced by molecular targeted GTB toxicity, which ultimately helps in ovarian cancer treatment. In future studies, xenograft tumor modeling, *in vivo* animal modeling, cell cycle analysis, *in vivo* mucoadhesive studies are warranted to know better optimization of the bio-adhesive intravesical delivery system for ovarian cancer

## Data Availability

The data that support the findings of this study are available from the corresponding author, [Sankha Bhattacharya], upon reasonable request.
